# Supplementary material for: Regional variation in knowledge and practice regarding common zoonoses among livestock farmers of selective districts in Nepal
Source: PLoS Negl Trop Dis. 2023 Feb 14;17(2):e0011082. doi: 10.1371/journal.pntd.0011082 (PMC9928098; doi:10.1371/journal.pntd.0011082)
Supplement: Attachment — Submitted filename: PNTD-D-22-00597_reviewer_comments.pdf [file pntd.0011082.s002.pdf]

PLOS Neglected Tropical Diseases

Regional variation in knowledge and practice regarding common zoonoses in livestock farmers of selective districts in Nepal

--Manuscript Draft--

**Manuscript Number:**	PNTD-D-22-00597
**Full Title:**	Regional variation in knowledge and practice regarding common zoonoses in livestock farmers of selective districts in Nepal
**Short Title:**	Regional variation in knowledge and practice on common zoonoses in livestock farmers
**Article Type:**	Research Article
**Keywords:**	Gandaki province; Livestock farming; Regional variation; Zoonoses
**Abstract:**	Abstracts<br><br>Background<br><br>As an agrarian country, the majority of the people in Nepal are involved in the farming profession. However, due to limited knowledge of zoonotic and poor preventive practices, vulnerabilities to zoonotic diseases are transmitted from livestock. The main objective of this study was to assess the zoonotic risk to livestock farmers based on their existing zoonotic-related knowledge and practices by applying the quantitative research design.<br><br>Methods<br><br>The total sample size was 380 from randomly selected three ecological zones of Nepal, and systematic sampling techniques were applied for data collection.<br><br>Result<br><br>Almost all respondents (91.8%) in the study adopted mixed types of livestock farming. Of the six zoonotic diseases, almost all (95.8%) respondents knew about zoonotic bird flu and rabies (90.7%), whereas only average respondents (54.2%) knew about swine flu. However, the very smallest number of respondents had known about bovine tuberculosis, neurocysticercosis, and brucellosis. Zoonoses preventive practices (i.e., handwashing with soap water 60.8%, mask 6.6%, gloves 1.8%, boots 1.3%, worn regularly) as a piece of personal protective equipment (PPE) provide empirical evidence of poor practices in the study. Not only that, 88% of respondents-built sheds/farms within 15 m. of their residents, 17% of households still consumed meat from sick animals, and vaccination of livestock also found poor coverage (36.0%) in the study.<br><br>Conclusion<br><br>Many common zoonotic diseases are poorly understood and practiced by livestock farmers in Nepal, with significant regional variation. So, to prevent zoonoses, we need to design interventional activities for livestock farmers based on the prevalence status of specific zoonoses in specific areas.
**Additional Information:**	
**Question**	**Response**
**Financial Disclosure**<br><br>Enter a financial disclosure statement that describes the sources of funding for the work included in this submission. Review the submission guidelines for detailed	No specific funding was received for this study.

Powered by Editorial Manager® and ProduXion Manager® from Aries Systems Corporation

requirements. View published research articles from *PLOS NTDs* for specific examples.<br><br>This statement is required for submission and **will appear in the published article** if the submission is accepted. Please make sure it is accurate.<br><br>**Unfunded studies**<br><br>Enter: *The author(s) received no specific funding for this work.*<br><br>**Funded studies**<br><br>Enter a statement with the following details:<br>• Initials of the authors who received each award<br>• Grant numbers awarded to each author<br>• The full name of each funder<br>• URL of each funder website<br>• Did the sponsors or funders play any role in the study design, data collection and analysis, decision to publish, or preparation of the manuscript?<br>• **NO** - Include this sentence at the end of your statement: *The funders had no role in study design, data collection and analysis, decision to publish, or preparation of the manuscript.*<br>• **YES** - Specify the role(s) played.<br><br>* typeset	
**Competing Interests**<br><br>Use the instructions below to enter a competing interest statement for this submission. On behalf of all authors, disclose any competing interests that could be perceived to bias this work—acknowledging all financial support and any other relevant financial or non-	we have not any funds for this research

financial competing interests.<br><br>This statement **will appear in the published article** if the submission is accepted. Please make sure it is accurate. View published research articles from *PLOS NTDs* for specific examples.<br><br>**NO authors have competing interests**<br><br>Enter: *The authors have declared that no competing interests exist.*<br><br>**Authors with competing interests**<br><br>Enter competing interest details beginning with this statement:<br><br>*I have read the journal's policy and the authors of this manuscript have the following competing interests: [insert competing interests here]*<br><br>* typeset<br><br>This statement is required for submission and **will appear in the published article** if the submission is accepted. Please make sure it is accurate and that any funding sources listed in your Funding Information later in the submission form are also declared in your Financial Disclosure statement.	
**Data Availability**	No - some restrictions will apply

Authors are required to make all data underlying the findings described fully available, without restriction, and from the time of publication. PLOS allows rare exceptions to address legal and ethical concerns. See the PLOS Data Policy and FAQ for detailed information.<br><br>A Data Availability Statement describing where the data can be found is required at submission. Your answers to this question constitute the Data Availability Statement and **will be published in the article**, if accepted.<br><br>**Important:** Stating 'data available on request from the author' is not sufficient. If your data are only available upon request, select 'No' for the first question and explain your exceptional situation in the text box.<br><br>Do the authors confirm that all data underlying the findings described in their manuscript are fully available without restriction?	
**Describe where the data may be found in full sentences. If you are copying our sample text, replace any instances of XXX with the appropriate details.**<br><br>• If the data are **held or will be held in a public repository**, include URLs, accession numbers or DOIs. If this information will only be available after acceptance, indicate this by ticking the box below. For example: *All XXX files are available from the XXX database (accession number(s) XXX, XXX.)*.<br>• If the data are all contained **within the**	First-hand original data was used in this article.

Powered by Editorial Manager® and ProduXion Manager® from Aries Systems Corporation

**manuscript and/or Supporting Information files**, enter the following: *All relevant data are within the manuscript and its Supporting Information files.*<br>• If neither of these applies but you are able to provide **details of access elsewhere**, with or without limitations, please do so. For example:<br><br>*Data cannot be shared publicly because of [XXX]. Data are available from the XXX Institutional Data Access / Ethics Committee (contact via XXX) for researchers who meet the criteria for access to confidential data.*<br><br>*The data underlying the results presented in the study are available from (include the name of the third party and contact information or URL).*<br>• This text is appropriate if the data are owned by a third party and authors do not have permission to share the data.<br><br>* typeset	
Additional data availability information:	

Powered by Editorial Manager® and ProduXion Manager® from Aries Systems Corporation

Manuscript

Click here to access/download;Manuscript;Manuscript for submission.docx

Regional variation in knowledge and practice regarding common zoonoses in livestock farmers of selective districts in Nepal

Kosh Bilash Bagale[1], Ramesh Adhikari[2], Devaraj Acharya[3]

Abstracts

Background: As an agrarian country, the majority of the people in Nepal are involved in the farming profession. However, due to limited knowledge of zoonotic and poor preventive practices, vulnerabilities to zoonotic diseases are transmitted from livestock. The main objective of this study was to assess the zoonotic risk to livestock farmers based on their existing zoonotic-related knowledge and practices by applying the quantitative research design.

Methods: The total sample size was 380 from randomly selected three ecological zones of Nepal, and systematic sampling techniques were applied for data collection.

Result: Almost all respondents (91.8%) in the study adopted mixed types of livestock farming. Of the six zoonotic diseases, almost all (95.8%) respondents knew about zoonotic bird flu and rabies (90.7%), whereas only average respondents (54.2%) knew about swine flu. However, the very smallest number of respondents had known about bovine tuberculosis, neurocysticercosis, and brucellosis. Zoonoses preventive practices (i.e., handwashing with soap water 60.8%, mask 6.6%, gloves 1.8%, boots 1.3%, worn regularly) as a piece of personal protective equipment (PPE) provide empirical evidence of poor practices in the study. Not only that, 88% of respondents-built sheds/farms within 15 m. of their residents, 17% of households still consumed meat from sick animals, and vaccination of livestock also found poor coverage (36.0%) in the study.

Conclusion: Many common zoonotic diseases are poorly understood and practiced by livestock farmers in Nepal, with significant regional variation. So, to prevent zoonoses, we need to design interventional activities for livestock farmers based on the prevalence status of specific zoonoses in specific areas.

Keywords: Gandaki province; Livestock farming; Regional variation; Zoonoses

Introduction

Agriculture is the prime source of the country's economy, which equally contributes to the livelihoods of the farming communities and has a significant impact on the national Gross Domestic Product (GDP). According to an economic survey published by the ministry of finance, it is estimated that in the fiscal years 2020 /021, out of total GDP, 20.2 percent of the contribution is covered by the agriculture sector [1]. In Nepal, approximately 66% of the population is still engaged in agriculture and livestock farming as a profession [2], with the majority of these being mixed types of livestock farming, including poultry and buffalo. Based on ecological diversity, livestock farmers in Nepal adopt different types of livestock in their households. Farmers in the Mountains keep Yak / Chauri and sheep whereas farmers in the Hills rear cows, sheep, goats, and poultry, and in Terai, buffalo, cows, goats, and poultry are the major domesticated livestock.

Due to globalization, the industrialization and commercialization paradigms are shifting in the field of livestock farming as a commercial profession. However, they are still adopting traditional and vulnerable farming practices with low knowledge of livestock (vertebrate animals) that transmit human illnesses called zoonoses [3]. Not only that, they use fertilizers, pesticides, or antibiotics for their agro-farming and

livestock to get higher production, but they are ignoring their hazardous health implications, including antibiotic resistance. Without proper knowledge of safety and technical guidance, they are facing several vulnerabilities, including zoonotic diseases and huge human and economic losses.

According to Taylor and colleagues, animal sources cause more than 60% of infectious diseases in humans [4]. People who work with or near animals (livestock) are thus more vulnerable to zoonotic infections, which can be fatal. But people are ignoring such realities and also neglecting issues in the country. In Nepal, many zoonotic diseases are prevalent in different forms, i.e., sporadic, endemic, epidemic, and so on [5]. When they get a favorable environment, that may change to a pandemic nature. So, to prevent zoonoses, we need to make knowledgeable at least those people who are close to and caretakers of their livestock (i.e., livestock farmers) by several interventional approaches (i.e., education or training). However, due to limited studies in the field of livestock farming, we have no actual ideas about the knowledge, and practices of livestock farmers related to zoonotic diseases and preventive practices. So, this study aims to identify the realities of knowledge and existing preventive practices of livestock farmers in the different ecological zones of Gandaki province in Nepal.

Materials and methods

This study followed a cross-sectional quantitative research design with a descriptive nature. Respondents were selected by systematic random sampling technique from randomly selected three ecological zones namely; Manang, Tanahun, and Nawalpur districts of Gandaki province in Nepal.

Population and sample size

Farmers who were keeping any types of livestock (including poultry and buffalo) and actively involved in livestock care roles were the population of this study. Based on the national population and housing census 2011, the total number of households (livestock farmers), including in all study districts (municipals), was 2835. The sample size in the study was estimated using the probability proportional to size (PPS) sampling technique proposed by Solvin [6], and the total sample size, including 10% for non-response rate, was 390.

Data collection tools, techniques, and analysis procedures

This study was a part of the Ph.D. of the first author. After taking the approval letter from GSE-TU, visited the respective municipalities and took the official approval for data collection. Researchers spent almost 74 days including all study districts for data collection, and a total of 380 respondents took the survey interview with face-to-face techniques in their households. In the preliminary phase of the data collection procedure, we established a good rapport with the participants and then explained the research objectives. Standardized tools were used for data collection, which was designed after being reviewed and discussed by experts as a Delphi technique and pre-tested. The questionnaire is divided into four sections: (i) respondents' socio-demographic status; (ii) knowledge about zoonoses; (iii) zoonoses preventive practice; and (iv) perception measuring questionnaire (perception related parts are not included in the article). According to the nature of the data, collected data was analyzed using descriptive statistics, including bivariate analysis.

Ethical consideration

The study proposal was approved by the research committee board of the Graduate School of Education, Tribhuvan University, Nepal. During the data collection, the ethical standard was maintained as per Nepal Health Research Council (NHRC) guidelines [7]. Verbal consent was taken before the interview and was requested to participate voluntarily. All data were kept confidential with anonymity. Moreover, we also followed the ethical guidelines made by the American Psychological Association (APA) [8] throughout the research process.

Results

This study was conducted in the Manang, Tanahun, and Nawalpur districts which are located in Gandaki province and represent all three ecological zone (Mountain, Hill, and Terai) of Nepal. Among the respondents, 32 (8.4%) were from Manang, 176 (46.3%) were from Tanahun, and 172 (45.2%) were covered by Nawalpur district as probability proportional to size (PPS) sampling technique.

Table 1. Socio-demographic characteristics of the respondents

Variables	Attributes	Frequency	Percent
District	Manang	32	8.4
	Tanahun	176	46.3
	Nawalpur	172	45.2
Gender	Female	174	45.8
	Male	206	54.2
Age group	20 -39	227	59.7
	40 -59	128	33.7
	60 and above	25	6.6
Main occupation of household	Livestock/ Poultry	30	7.9

	Agro farming	257	67.6
	Service	33	8.7
	Foreign employee	29	7.6
	Trade	27	7.1
	Other	4	1.1
Educational status of respondents	Illiterate	16	4.2
	Up to basic level	199	52.4
	Secondary and above	165	43.4
Aim of livestock farming	Household consuming	328	86.3
	Commercial farming	52	13.7
Training related to farming	Yes (short course)	11	2.9
	No	369	97.1
Type of livestock farming	Single	31	8.2
	Mixed farming	349	91.8
Keeping livestock in the household +	Cow	107	28.2
	Buffalo	200	52.6
	Goat/sheep	260	68.4
	Pig	26	7.1
	Poultry	340	89.5
	Yak/Chauri	7	1.8
	Other	8	2.1

+ Percentage may exceed 100 due to multiple responses

The majority (54.2%) of the respondents in the study were male, nearly 3 in 5 (59.7%) were between 20 to 39 years and the median age of the respondents was 35 years. More than two-thirds (68%) of respondents adopted agro-farming and 7.9 percent were dependent on livestock farming as the main occupation in their households. More than half (52.0%) had basic level education, and very nominal (3.0%) of livestock farmers got training related to livestock farming. Likewise, almost respondents (91.8%) were keeping

mixed types of livestock in their household, where poultry, goat/ sheep, and buffalo are highly keeping livestock (90%, 68%, and 53% respectively) in their household and the main purpose of that farming was household consumption (86%).

Knowledge of common zoonoses based on ecological zone

Rabies: almost respondents (91%, n=380) had heard about zoonotic rabies. However, higher respondents in Nawalpur (95.3%, n=172) had known about zoonotic rabies than in the other two districts which seem significant association (p=.007) between zoonotic rabies-related knowledge based on the different ecological zone in the study.

Table 2. Rabies; knowledge-based on ecological zone

Variables	Total		Manang		Tanahun		Nawalpur		P-value
	N=380	%	N=32	%	N=176	%	N=172	%	
Heard	345	90.7	30	93.8	151	85.8	164	95.3	.007
No heard	35	9.2	2	6.2	25	14.2	8	4.7	
Causative agent of rabies									
Virus*	115	33.33	3	10.0	24	15.9	88	53.7	.000
Others	91	26.37	2	6.7	76	50.3	13	7.9	
I don’t know	139	40.2	25	83.3	51	33.8	63	38.4	
Symptoms of rabies +									
Hydrophobia	302	87.5	11	36.7	148	98.0	143	87.2	.000
Salivation	145	40.02	19	63.3	17	11.3	109	66.5	.000
Irritation	59	17.1	8	26.7	13	8.6	38	23.2	.000
Rabies mode of transmission									
Bitten by rabid animals... *	319	92.4	27	90.0	136	90.1	156	95.1	.000
Other	15	4.3	0	0	15	9.9	0	0	
I don’t know	11	12.1	3	10.0	0	0.0	8	4.9	

Preventive Measure of rabies									
Wash the wound and contact in HFs *	336	97.4	25	83.3	148	98.0	163	99.4	.000
Others	4	1.1	1	3.3	3	2.0	0	0	
I don’t know	5	1.4	4	13.3	0	0.0	1	0.6	
Knowledge of ARV (Freely availability in Gov. hospital)									
I know	153	44.3	0	0	40	26.5	113	68.9	.000
I don’t know	192	55.7	30	100.0	111	73.5	51	31.1	

+ Percentage may exceed 100 due to multiple responses// * correct answer

Similarly, among the total respondents, one-third (33.3%) were known about the causative agent of rabies with higher respondents in Nawalpur (53.7%, n=172) than in Manang (10.0%, n=32) and Tanahun (16.0%, n=176). However, respondents had good knowledge of symptoms of rabies (hydrophobia) by 98.0 and 87.2 percent respondents in Tanahun and Nawalpur than the symptoms of salivation (11.3% and 66.5%) and irritation (8.6% and 23.2%) respectively. But, in Manang greater number of the respondents knew about symptoms of salivation (63.3%) than hydrophobia and irritation (36.7% and 26.7%).

Bitten by rabid animals or contact saliva in an open wound is the mode of transmission of the rabies virus. Knowledge of these variables was found satisfactory in all districts. Where, 90.0, 91.1, and 95.1 percent of respondents knew Manang, Tanahun, and Nawalpur respectively and similar knowledge was also found in the preventive measures of rabies in all study districts. Where higher respondents in Nawalpur had a piece of knowledge on preventive methods (99.4%) to compare the Tanahun (98%) and Manang (83%). However, knowledge on the free availability of anti-rabies vaccine (ARV) up to primary health center (PHC) found poor knowledge (0, 26.5, and 68.9%) in

Manang, Tanahun, and Nawalpur respectively in the study, and all variables related to knowledge found significant association (p<.001) with the ecological zone of the study.

Bird flu: it is a highly known zoonotic disease among the respondents and also highly infectious in avians. However, due to close exposure without any safety people will get bird flu from their poultry which may be dangerous to the victim.

Table 3. Bird flu; knowledge-based on ecological zone

	Total		Manang		Tanahun		Nawalpur		
Variables	N=380	%	N=32	%	N=176	%	N=172	%	P-value
Heard about Bird flu	364	95.8	28	87.5	173	98.3	163	94.8	.013
No Heard	16	4.2	4	12.5	3	1.7	9	5.2	
Causative Agent of Bird flu									
Virus*	226	62.1	9	32.1	123	71.1	94	57.7	.000
Others	11	3.0	0	0	5	2.9	6	3.7	
I don't know	127	34.9	19	67.9	45	26.0	63	38.7	
Bird flu Mode of Transmission +									
Contact without safety….	283	77.7	12	42.9	124	71.7	147	90.2	.000
Consumed raw poultry product	349	95.9	25	89.3	171	98.8	153	93.9	.000
Contact infected poultry	81	22.2	12	42.9	21	12.1	48	29.4	.000
Symptoms of Bird flu in Poultry +									
Diarrhea	124	34.1	3	10.7	105	60.7	16	9.8	.000
Nasal discharge	51	14.0	3	10.7	34	19.7	14	8.6	.027
Cough sneezing	21	5.8	2	7.1	13	7.5	6	3.7	.303
Swelling eyelid and joint……	224	61.5	3	10.7	159	91.9	62	38.0	.000
Preventive Measures of Bird flu +									

Consumed well-cooked poultry…	345	94.8	22	78.6	172	99.4	151	92.6	.000
Handwashing with soap	286	78.6	22	78.6	143	82.7	122	74.8	.141
Use PPE	153	42.1	7	25.0	40	23.1	106	65.0	.000

+ Percentage may exceed 100 due to multiple response // * correct response

Almost respondents in the study (96%) were heard about zoonotic bird flu. However, to compare the studies districts; higher respondents in Tanahun had a higher knowledge (98.3%) than in Nawalpur (94.8%), and Manang (87.5%). But, knowledge of causative agents was found lower to compare the previous variable (heard about bird flu) in the study. Ecologically, only 71, 58, and 32 percent of respondents knew with higher variation in Tanahun Nawalpur and Manang respectively with statistically significant (p<.001) in the study.

If people have information about the way of disease transmission, they will break this chain which helps to protect from infection. In the case of bird flu, results were found satisfactory in the study. Respondents in all study districts had a higher knowledge of bird flu, it is transmitted due to consumed raw poultry products (meat or egg) with regional variation (p<.001). However, symptoms of bird flu in poultry found poor knowledge among the respondents in Manang and Nawalpur to compare the Tanahun. Where 7.1 to 10.7 percent of respondents in Manang, 7.5 to 91.9 percent in Tanahun, and 3.7 to 38 percent of the respondent in Nawalpur had at least knowledge of any symptoms of bird flu in poultry with statistically significant (p<.001 to .027). Similarly, respondents had a good knowledge of preventive measures for bird flu in Tanahun to compare the other two districts.

Swine flu: it is a highly contagious zoonotic disease transmitted by an infected pig (swine). People who are close to infected pigs without safety will be vulnerable to swine

flu. So, farmers, who have a swine farm most have a basic knowledge about the pig-related zoonoses.

Table 4. Swine flu; knowledge-based on ecological zone

Variables	Total		Manang		Tanahun		Nawalpur		
	N=380	%	N=32	%	N=176	%	N=172	%	P-value
Heard	206	54.2	7	21.9	93	52.8	106	61.6	.000
No heard	174	45.8	25	78.1	83	47.2	66	38.4	
Causative agent of swine flu									
Virus*	121	58.7	3	42.9	67	72.0	51	48.1	.000
Others	19	9.2	0	0	16	17.2	3	2.8	
I don't know	66	32.1	4	57.1	10	10.8	52	49.1	
Symptoms of swine flu									
Like a normal flu	160	77.7	4	57.1	89	95.7	67	63.2	.000
Others	3	1.4	0	0	1	1.1	2	2.1	
I don't know	43	20.9	3	42.9	3	3.2	37	34.9	
Swine flu mode of transmission									
Droplet infection	160	77.7	4	57.1	89	95.7	67	63.2	.000
Other	2	0.9	0	0.0	1	1.1	1	0.9	
I don't know	44	21.3	3	42.9	3	3.2	38	35.8	
Preventive measures of swine flu +									
Don't touch the facial part	160	77.7	5	71.4	88	94.6	67	63.2	.001
Avoid travel unnecessarily	36	17.5	3	42.9	10	10.8	23	21.7	.050
wear mask	123	59.7	5	71.4	55	59.1	63	59.4	.060

+ Percentage may exceed 100 due to multiple response // * correct response

In this study, more than half (54%, n=380) of respondents have heard about swine flu however significant number of respondents had unknown of it. Ecologically, higher respondents in Nawalpur (61.6%) were known about swine flu than in Tanahun (52.8%) and Manang (21.9%) with significant regional variation (p<.001). Similarly, more than half (58.7%, n=206) of respondents knew the causative agent of zoonotic swine flu. Ecologically, more respondents (72%) in Tanahun than in Nawalpur (48%) and in Manang (43%) had a piece of knowledge on the causative agent (p<.001).

Variables on symptoms and mode of transmission of swine flu found similar knowledge in the study. However, the highest respondents (95.7%) knew Tanahun to compare Nawalpur (63.2%) and Manang (57.1%) with significant regional variation (p<.001). Mask wearing is the preventive technique for swine flu also response likely similar respondents in all study districts. Whereas, don't touch facial part unnecessarily response by higher respondents in Tanahun (95%) than in Manang (71%) and Nawalpur (63%, p<.001). However, avoiding unnecessary travel was the response by the least respondents in the study.

Out of the studied six common zoonotic diseases, very least respondents knew about zoonotic brucellosis, neurocysticercosis, and bovine tuberculosis, so we present data very briefly in the study.

Brucellosis: is a common zoonotic disease in Nepal. It is transmitted commonly from aborted livestock and contaminated milk and milk production. However, very least (1.6%, n=380) respondents were known about zoonotic brucellosis in the study. Ecologically, no one respondents in Manang and equally (1.8%, n=176) in Tanahun and Nawalur (1.8%, n=172) were heard about brucellosis. So, brucellosis had a low knowledge among the respondents in all study districts.

Neurocysticercosis (NCC): it is a zoonotic disease or condition which occurs when a cyst of tapeworm travel in the human brain. It is a common zoonotic in Nepal however, very few respondents (2.7%, n=380) in the study knew NCC. Ecologically, higher respondents in Manang (13%, n=32) than in Tananhu (1.7%, n=176) and Nawalpur (1.8%, n=172) had heard about it with significant regional variation (p<.001).

Bovine tuberculosis: is also a common zoonotic disease in Nepal. People who are close to cattle without any safety are vulnerable to bovine tuberculosis. However, very least respondents (3.1%, n=380) knew about bovine TB. To compare the study districts higher respondents in Nawalpur (5.2%, n=172) had heard about BTB than in Manang (0.0) and Tanahun (1.7%, n=176).

So, based on data, respondents had a good knowledge of zoonotic bird flu and rabies whereas swine flu had average knowledge. However, bovine tuberculosis, neurocysticercosis, and brucellosis found the very least knowledge with significant regional variation in the study.

Zoonoses preventive practices based on ecological zone

Healthy practices determine positive health and well-being. Due to the host and reservoir characteristics of animals, livestock farmers who are close exposure to the livestock are facing vulnerabilities related to zoonoses. So, to protect from zoonoses we need to follow safety practices during the take care/close exposure to the livestock. However, in this study, only more than half (60.8%, n=380) of respondents had regularly washed their hands with soap water after close exposure to livestock. Ecologically, higher respondents (91.9%, n=172) in Nawalpur regularly wash their hands with soap water than in Manang (71.9%, n=32) and Tanahun (28.4%. n=176). Mask-wearing practices during close exposure also found poor practices in the study, where only 25 (6.6%) livestock

farmers wear masks regularly but due to the impact of COVID 19 pandemic 42 percent of farmers used masks occasionally. To compare the results in the studied three districts, respondents in Nawalpur found higher regular mask users (12%, p<.001) with significant regional variation (Table 5).

Table 5. Practices of PPE by livestock farmers to prevent zoonoses

Variables	Frequencies		Manang		Tanahun		Nawalpur		P-value
Handwashing with soap water after close contact with livestock									
	N=380	%	N=32	%	N=176	%	N=172	%	
Regular	231	60.8	23	71.9	50	28.4	158	91.9	.564
Occasionally	149	39.2	9	28.1	126	71.6	14	8.1	
Mask wearing during exposure to livestock/poultry									
Regular	25	6.6	2	6.3	2	1.1	21	12.2	.000
Occasionally	161	42.4	4	12.5	107	60.8	50	29.1	
Never	194	51.1	26	81.3	67	38.1	101	58.7	
Gloves used during caring the livestock/poultry									
Regular	7	1.8	1	3.1	1	0.6	5	2.9	.000
Occasionally	106	27.9	15	46.9	71	40.3	20	11.6	
Never	267	70.3	16	50.0	104	59.1	147	85.5	
Boot wearing during exposure to livestock/poultry									
Regular	5	1.3	2	6.3	0	0	3	1.7	.000
Occasionally	77	20.3	19	59.4	46	26.1	12	7.0	
Never	298	78.4	11	34.4	130	73.9	157	91.3	
Availability of the above PPE in households during interview time									
Soap	347	91.3	32	100	167	94.9	148	86.0	
Mask	150	39.4	5	15.6	64	36.4	81	47.1	
Gloves	56	14.8	10	31.3	19	10.8	27	15.7	
Boots	31	8.1	20	62.5	5	2.8	6	3	

Similarly, only 7 (1.8%) respondents used gloves regularly. Ecologically, respondents in Manang found higher regular gloves users (3.1%) than in the other two districts during close exposure to livestock. Likewise, boot-wearing practices also found similar practices (1.3%, n=380) among the respondents with the higher regular user in Manang (6.3%, n=32) with significant regional variation (p<.001). Those practices indicate that livestock farmers are facing zoonoses-related vulnerability due to a lack of properly used PPE. Hand washing practices which are easy and affordable PPE in farming communities also found poor practices in the study. However, the proportion between using practices and availability of PPE in the household (like; soap, mask, gloves, and boots) during the interview period was found reliable.

Most of the respondents practiced mixed types of livestock farming with traditional practices. Economic status, education, or several socio-cultural values might determine farm and household sanitation in farming communities. Most of the respondents (88%, n=380) in the study build livestock sheds/farms near than 15 meters from their residents (Table. 6). From the perspective of public health standard distance between home and shed is more than 15 meters or 50 fits [9]. Based on ecology, almost respondents in Manang (91%, n=32) maintain this standard than in Nawalpur (9%, n=172) and Tanahun (1%, n=176) with significant regional variation (p<.001).

Table 6. Livestock-related common practices in farming communities

Variables	Frequencies		Manang		Tanahun		Nawalpur		*P*-value
	N=380	%	N=32	%	N=176	%	N=172	%	
Distance between Home and Shed									
Less than 15 M.	334	87.9	3	9.4	174	98.9	157	91.3	.000

More than 15 M.	46	12.1	29	90.6	2	1.1	15	8.7	
Disposal practices of dead animals									
Borehole	336	88.4	6	18.8	176	100.	154	89.5	.000
Throw the river	44	11.6	26	81.2	0	0	18	10.5	
Children exposure to livestock									
Yes	254	66.8	28	87.5	149	84.7	77	44.8	.000
No	126	33.2	4	12.5	27	15.3	95	55.2	
Pregnant women exposure to livestock									
Yes	248	65.3	31	96.9	153	86.9	64	37.2	.000
No	132	34.7	1	3.1	23	13.1	108	62.8	
Sick animal consuming practices									
Yes	64	16.8	23	71.9	17	9.7	24	14.0	.000
No	316	83.2	9	28.1	159	90.3	148	86.0	
Vaccination practice for livestock									
Yes	135	35.5	3	9.4	8	4.6	124	72.1	.000
No	245	64.5	29	90.6	168	95.4	48	27.9	

M= meter

Similarly, almost respondents (88.4%) dispose of dead animals in a borehole whereas 12 percent throw the nearest river. These practices were found best (100%) among the respondents in Tanahun than in Nawalpur (89%) and Manang (19%). Due to low immunity children and pregnant period are more vulnerable to zoonotic transmission. However, more than two-thirds (67%) of respondents said that their children were close exposure to livestock. Those exposures were found higher in Manang and Tanahun than in Nawalpur (88, 85, and 45%) respectively. Likewise, more than two-thirds (65%) of households' pregnant women were close exposure to their livestock as a role taking care. This practice was found higher in Manang (97%) and Tanahun (87%) than in Nawalpur (37%). Likewise, 64 (17%) households still consumed the meat of sick animals. This

practice was found higher in Manang (72%) than in Nawalpur (14%) and Tanahun (10%). Vaccination practices for their livestock also found poor coverage in the study. Out of the total respondents' farmers, only two-thirds (36%) were vaccinated their livestock. To compare this practice, Nawalpur had the highest coverage (72%) than Manang (9%) and Tanahun (5%). Based on data majority of the farmers ignored vaccination to livestock as pre-exposure prophylaxis in all ecological zones which may induce zoonoses vulnerabilities to the farming communities and all preventative practices found significant regional variation ($p<.001$) in the study.

Discussion

As an agrarian country, a large number of people in Nepal are involved in the field of agro or livestock farming [2], and farmers are exposed to zoonotic agents in every aspect of their work. Several studies have found that many zoonotic diseases are prevalent in Nepal in various forms (sporadic, endemic, epidemic, and so on) [[illegible]]. So, people who have close exposure to livestock or related professions need to be knowledgeable of livestock-related common zoonoses which are prevalent in their territory.

In this study, we found that avian influenza was the mostly known common zoonotic disease among livestock farmers, followed by rabies and swine flu. However, bovine tuberculosis, neurocysticercosis, and brucellosis had the very least knowledge, respectively. All studied zoonotic diseases have endemic potential in Nepal [10] and have a direct impact on the health of livestock and caretaker farmers. Symptoms look similar to many other human illnesses with low case fatalities, farmers may ignore zoonotic diseases like brucellosis, bovine tuberculosis, and neurocysticercosis in the study.

Knowledge compares on studied zoonoses among the livestock farmers in all three ecological zones, higher respondents in Nawalpur knew about zoonotic rabies, swine flu, bovine tuberculosis, and brucellosis. However, brucellosis and bovine tuberculosis had the very least knowledge to Nawalpur. Whereas, respondents in Tanahun found higher knowledge of avian influenza and neurocysticercosis in Manang.

Rabies is a life-threatening zoonotic disease that has endemic potential in many parts of developing countries like Nepal. Due to close contact with livestock without any safety and the burden of stray dogs, almost half of the Nepalese population is at risk and a quarter at moderate risk of rabies [11]. Most respondents in the study heard about zoonotic rabies with regional variation, which was higher than among livestock farmers in Punjab, India [12].

Results compared among the study districts on zoonotic rabies, more respondents in Nawalpur responded with the correct answer on causative agents, mode of transmission, preventive measures, and free ARV services (it is supply based on caseload and institutional demand order) up to the PHC and district level government hospital in the study. However, more respondents in Tanahun responded with the correct answer to the symptoms of rabies. Those results were similar to livestock farmers in Punjab, India [12] and traditional farmers in Jimma, Southwestern Ethiopia, where (82.8%; n = 75) respondents perceived the prognosis of rabies as that of a person who was bitten by a rabid dog and had not gotten ARV would die [13], which was higher knowledge than in this study.

As a health educator data analyzed with a critical perspective, rabies is a fatal disease if the victim does not take an ARV as post-exposure prophylaxis (PEP), however, knowledge of ARV was not satisfactory in this study. Due to poor knowledge of disease

fatalities and free ARV services, people may not perceive disease threats and refuse ARV, which contributes to the rabies burden in the Nepalese community and also creates a challenge to achieving rabies eradication by 2030 in Nepal [14].

Similarly, avian influenza is mostly known as a zoonotic disease in all studied districts. Due to frequent outbreaks [in Nepal, avian influenza was first seen in 2009, after it was seen 237 times in different parts of the country up to fiscal years 2016/17 and 1,966,745 poultry were slaughtered [15], most of the respondents in all studied districts heard about avian influenza. However, more respondents in Tanahun responded with the correct answer when compared to the other two districts. Out of five knowledge-measuring questionnaires, more respondents in Tanahun responded with the correct answer on having heard of the bird flu, causes, and signs/symptoms, but more respondents in Nawalpur responded with the correct answer in the mode of transmission and preventive measures. This result was similar to livestock farmers in Punjab, India [12] where 92.4 percent of farmers knew about avian influenza.

Swine flu is a highly contagious disease with a pandemic nature. In 2009, a pandemic outbreak began in Mexico [16]. People who are involved in pig farming or anyone who has close exposure to pigs and related professions is more vulnerable to swine flu. In this study, only more than half of the respondents had heard about swine flu, which indicates vulnerability to zoonotic swine flu in farming communities. To compare the study of three districts, more respondents in Nawalpur had knowledge of swine flu than in Tanahun and Manang. This result was very low compared with the study in Punjab, India, where almost all respondents had heard about swine flu [12]. So, for farmers who are close to swine, this type of knowledge will help to protect against several zoonotic diseases, including swine fever.

Similarly, very few respondents (3.1%) had heard about bovine tuberculosis (BTB), with higher respondents in Nawalpur responding to the correct answer than in the other two districts. However, the percentage of correct responses in all variables was found very low in all study districts. It was poor knowledge of the livestock farmers in Punjab, India [12], smallholder dairy farmers in North Shewa, Ethiopia [16], and community people in Ghana [17].

It is a cattle-related common zoonosis, but most livestock farmers in Nepal have little or no knowledge of bovine tuberculosis, which poses a challenge not only for veterinarians but also for public health and community members. A study conducted in Chitwan, Nepal, found that in 60 TB patients' households, 15 percent of livestock were found to be BTB, and all TB patients were involved in feeding, milking, and other roles of taking care of livestock, and one-fourth of patients' habits of raw milk consumption [18]. Those types of practices are vulnerable to further disease transmission in the community. Another study showed that in households with 50 cattle, after the intradermal cervical tuberculin test, 10 percent of animals were found to have Mycobacterium bovis [19]. However, due to a lack of proper study, we had no data on the proportion of human tuberculosis cases from bovine sources. However, it is believed that most of the extrapulmonary (EP) tuberculosis cases in humans are from bovine sources [20].

Brucellosis is the second most important disease in the world after rabies, and it has been reported as endemic in Nepal [21]. Respondents had the least knowledge about brucellosis, with only 1.6 percent having heard about it, which was very low in comparison to the finding [22] in Tajikistan, where 15.0 percent of respondents had heard about brucellosis, and a similar finding found in Shewa, Ethiopia [23], but none

of the respondents had heard of such a disease as brucellosis in South-western Ethiopia [13]. In comparison, 88 percent of farmers in Kars, Turkey, were aware of brucellosis [24], and livestock farmers in Punjab were also aware of the disease [12]. This study also proved that brucellosis-related knowledge in respondents seems associated with the economic and educational status of the countries, which was proved to compare the studies on Punjab, India, Kars, Turkey, Ethiopia, and Nepal.

In the same way, knowledge of neurocysticercosis was low (2.7%) compared to the study in Southwestern Ethiopia, where two-thirds of the traditional farmers (n = 48) knew taeniasis. They also shared that taeniasis is transmitted to humans when raw meat [Taenia saginata (beef tapeworm), Taenia solium (pork tapeworm)] is consumed [13], but more than two-thirds of traditional farmers had a habit of raw meat consumption, and among the raw meat consumers, almost all have been infected by taenia saginata at least once previously. Several patient reports from Asian countries indicate the wide prevalence of taenia solium cysticercosis, and it is the major cause of epilepsy in Indonesia, Vietnam, and Nepal [25]. When compared with the other three districts, respondents in Manang found higher correct answers in all knowledge measuring questionnaires (3.1–12.5%) than in other districts.

Human behaviors and practices are influenced by their existing knowledge, socio-cultural values, or perceptions in a particular context. In the context of Nepal, most livestock farmers follow the traditional practices, adopting mixed types of livestock farming and exposing themselves to safety. However, due to host and reservoir characteristics of several lethal pathogens, caretaker farmers are facing susceptibilities to zoonotic diseases in their keeping livestock.

As a care taker, livestock farmers need to take some precautions to prevent zoonotic diseases. Hand washing, mask use, gloves, and boots are some of the low-cost and effective practices for preventing several highly contagious zoonotic diseases. However, only more than half (60.8%) of respondents in this study wash their hands with soap and water on a regular basis after close exposure to their livestock, and similar practices were found in all ecological zones in the study. In contrast, in a study in Chitwan, Gorkha, and Tanahun districts, a higher number (94.0%) of smallholder farmers wash their hands with soap water after handling livestock [26], and in a study in the suburban areas of Bangladesh, where (100.0%) of smallholder livestock farmers wash their hands with soap water after interaction with animals [27]. Those results found an association with several motivational and interventional activities by the research project to compare with the current study.

Mask wearing during close exposure to livestock was also found to be poor practice in the study, where only seven percent of respondents used masks regularly. However, due to the impact of the threat perception of the COVID-19 pandemic, nearly half were used occasionally in the study. Ecologically, the very worst scenario was found in Tanahun (1%) than in Manang (6%) and Nawalpur (12%), and this finding was found similar to farmers in Kars, Turkey [24], where equal respondents like this study (6.6%) used masks regularly, but 84% of farmers considered it necessary.

Gloves worn regularly during close exposure to their livestock were also found to be poor practices (1.8%) in the study. More than two-thirds of respondents never used gloves, and nearly one-third used gloves occasionally. Ecologically, respondents in Manang found higher (3.1%) regular users than in Nawalpur and Tanahun, which may create zoonotic threats for livestock farmers (i.e., brucellosis). This was poor

practice compared to the study in Kars, Turkey [24], where 35.8% of farmers used gloves regularly while 92.1% had a positive attitude. But, only a few (0.8%) cattle farmers in the Tamale, northern region of Ghana, used gloves while handling sick cattle [28].

Likewise, boot-wearing practices (including regular and occasionally) found satisfactory results in Manang (65.0%) than in Tanahun (26.1%) and Nawalpur (8.7%). When compared to the findings in Kars, Turkey [24], where 42.4 percent of respondents wore boots on a regular basis during the exposure period, with an 89.4 percent positive perception.

Due to the lack of properly used personal protective equipment [PPE], the findings of this study indicate zoonotic threats to livestock farmers. Hand washing practices, which are simple and inexpensive PPE in farming communities, were also found to be poor in the study, and data showed a significant association ($p < .001$) between zoonotic preventive practices (i.e., mask-wearing, glove-wearing, and boot-wearing) and the study's ecological zone. So, to increase the regular use of PPE, stakeholders should plan motivational or interventional activities in the farming communities of Nepal based on the OH concept.

This study revealed many risk factors related to conventional livestock farming practices in Nepal. Economic status, education, or several socio-cultural values determine farm and household sanitation in farming communities. In this study, most of the respondents (88.0%) built animal sheds/farms less than 15 meters from their residences, and similar practice was observed in Bangladesh [29]. But in another study in Bangladesh, nearly half (43.0%) of the farms were attached to their houses [27]. From the public health point of view, the standard distance between a house and a shed

is more than 15 meters or 50 feet [9], which is maintained only by a few (12%) respondents in the study.

In comparison to the other districts studied, nearly all respondents in Manang found it easier to keep the distance between home and shed than in Tanahun and Nawalpur, and there was a significant association ($p < .001$) with these practices. One aspect of distance maintenance in Dharapani, Manang was that residents restricted livestock entry into the residential area.

Several zoonotic diseases like rabies, brucellosis, avian influenza, swine flu, neurocysticercosis, bovine tuberculosis, etc. are considered to be potential zoonotic diseases in Nepal. Along with these diseases, several other zoonotic diseases exist that will be transmitted to humans mainly through direct contact during taking care or other close exposure without PPE to the infected animal. The majority of respondents in the study (88.0%) disposed of dead animals in the borehole. The remaining few were thrown into the nearest river, which found similar practices on smallholder farmers in Nepal [26] where 18 percent of them disposed of animal waste (like; placenta) on the ground, in water, or on a tree as a customary practice was found vulnerable to zoonotic transmission. In Tanahun, Nawalpur, and Manang, respectively, 100, 89, and 19 percent of respondents dispose of dead animals in boreholes. Those practices show a significant association with an ecological zone in the study ($p = .001$).

There were several influencing factors for close exposure to children and livestock. Either to minimize the parent's workload or as a normal refreshment activity, the children of the respondents were closed to livestock in the study. More than two-thirds of respondents in the study disclosed that their children had had close exposure to livestock. Ecologically, Nawalpur had more respondents who avoided those practices

(55.2%) than Tanahun (15.3%) and Manang (12.5%), with significant ecological variation ($p < .001$). Not only in Nepal, but similar practices are also recorded in other parts of the globe, mainly in poor or developing countries. Similar results (70.0%) were revealed in suburban areas of Bangladesh, where children of respondent farmers had close exposure to their animals [27]. This may impact the education and economic status of the livestock farmers, who are found similarly in Bangladesh and Nepal.

Not only children, but more than two-thirds (65%) of the respondents' farmers stated that pregnant women were also close to their livestock as caregivers. These practices were found higher in Manang than in other districts and showed a significant association based on the ecological zone in the study ($p<.001$). Due to poor immunity, those types of exposure create a higher vulnerability to the health of the mother, fetus, or younger children from zoonotic diseases.

The consumption practices of sick or recently dead animals are influenced by several socioeconomic statuses. However, some of the respondents in this study (17%) still consume those practicing in their communities. These practices were found to be more prevalent in Manang (72%), than in Tanahun (14%), and Nawalpur (9.7%), with a significant association with the study's ecological zone ($p < .001$). In contrast, a study in Kars, Turkey, revealed good knowledge related to sick and dead animals, where four out of five respondents knew that they should be buried deep and practiced by one-fifth [24].

Most communicable diseases (including zoonoses in livestock) will be controlled by a cost-effective single-dose vaccination. However, due to several obstacles, farmers are facing many losses in the field of livestock farming. In this study, vaccination practices were also found to have poor coverage among the respondents.

Only two-thirds of farmers vaccinated their livestock as pre-exposure prophylaxis. Vaccination practices for their livestock were found higher (78.26%) in suburban areas of Bangladesh [27] than in this study. To compare the practices, Nawalpur had the highest coverage (72%) than Manang (9%) and Tanahun (5%) and showed a significant association with the study's ecological zone ($p < .001$). The majority of the respondents in the study ignored vaccination as pre-exposure prophylaxis in all ecological zones, which may be responsible for zoonotic outbreaks anytime and anywhere.

Conclusion

As an agrarian country, majority of the people in Nepal are involved in the farming profession, and they are exposed to zoonotic agents in every aspect of their work. So, to prevent zoonoses, they must have the knowledge and skills to follow preventive practices. However, the majority of them had no good idea about many of the studied zoonotic diseases that are endemic in their territory, and we found a significant regional variation in knowledge and zoonotic disease preventive practices in the study. If we make the livestock farming profession safer from the perspective of zoonoses through several interventional activities, it is not only a contribution to healthy livestock and healthy farmers but also equally contributes to poverty eradication and support for national GDP.

Acknowledgments: We would like to thank the participants for their time and responses.

References

[1] Ministry of Finance, "Economic Survey 2020/21 Government of Nepal Ministry of Finance," p. 147, 2021.

[2] S. Adhikari, "Contribution of agriculture sector to the national economy in Nepal," *J. Agric. Environ.*, vol. 16, pp. 180–187, Jun. 2015, doi: 10.3126/AEJ.V16I0.19851.

[3] WHO, “Zoonoses: key facts,” 2020. https://www.who.int/news-room/fact-sheets/detail/zoonoses (accessed Oct. 31, 2021).

[4] L. H. Taylor, S. M. Latham, and M. E. Woolhouse, “Risk factors for human disease emergence.,” *Philos. Trans. R. Soc. B Biol. Sci.*, vol. 356, no. 1411, pp. 983–989, Jul. 2001, doi: 10.1098/RSTB.2001.0888.

[5] S. Thapa, “Recent status of major infectious diseases in Nepal,” 2013. [Online]. Available: https://www.researchgate.net/publication/303519298_Recent_status_of_major_infectious_diseases_in_Nepal.

[6] A. Susanti, R. A. A. Soemitro, H. Suprayitno, and V. Ratnasari, “Searching the appropriate minimum sample size calculation method for commuter train passenger travel behavior survey,” *J. Infrastruct. Facil. Asset Manag.*, vol. 1, no. 1, pp. 47–60, 2019, doi: 10.12962/jifam.v1i1.5232.

[7] NHRC, *National Ethical Guidelines For Health Research in Nepal And Standard Operating Procedures*, no. January. 2011.

[8] APA, “Publication manual of the American Psychological Association,” *Am. Psychol. Assoc. (7th ed).*, 2020, [Online]. Available: https://apastyle.apa.org/products/publication-manual-7th-edition.

[9] K. Park, *Park’s textbook of preventive and social medicine.*, 20th ed. M/s Banarsidas Bhanot, 2009.

[10] Asian Network for Sustainable Agriculture and Bioresources (ANSAB), “Baseline

survey and pre-KAP (knowledge, attitude and practice) study in Chitwan, Rupandehi and Banke districts for One Health Asia programme (OHAP) in Nepal," 2015. Accessed: Oct. 07, 2021. [Online]. Available: https://ansab.org.np/storage/product/ansab-ohap-pre-kap-baseline-report-1579846344.pdf.

[11] DoHS, "Annual report F/Y 2072/073. Department of health services," 2017. Accessed: Oct. 23, 2021. [Online]. Available: https://dohs.gov.np/annual-report-2072073-new-published/.

[12] J. S. Hundal, S. S. Sodhi, A. Gupta, J. Singh, and U. S. Chahal, "Awareness, knowledge, and risks of zoonotic diseases among livestock farmers in Punjab.," *Vet. world*, vol. 9, no. 2, pp. 186–18691, 2016, doi: 10.14202/vetworld.2015.186-191.

[13] D. Tesfaye, D. Fekede, W. Tigre, A. Regassa, and A. Fekadu, "Perception of the public on the common zoonotic diseases in Jimma, Southwestern Ethiopia," *Int. J. Med. Med. Sci.*, vol. 5, no. 6, pp. 279–285, Jun. 2013, doi: 10.5897/IJMMS2013.0931.

[14] W. SAARC, OIE, "Enhancing progress towards rabies elimination ' Zero by 30 ' in SAARC Region," no. June, 2019, [Online]. Available: https://www.saohnet.org/wp-content/uploads/2019/12/Enhancing-Progress-towards-Rabies-Elimination-Zero-by-30-in-SAARC-Region_V3.5_20191122.pdf.

[15] K. P. Acharya, N. Acharya, S. Phuyal, and S. H. Subramanya, "Human infection with Avian influenza A virus in Nepal: requisite for timely management and preparedness," *VirusDisease*, vol. 31, no. 3, p. 244, Sep. 2020, doi: 10.1007/S13337-020-00593-Z.

[16] B. R. Adhikari, G. Shakya, K. P. KC, S. D. Shrestha, G. R. Upadhyay, and B. P. Dhungana, "Outbreak of pandemic influenza A/H1N1 2009 in Nepal," *Virol. J. 2011 81*, vol. 8, no. 1, pp. 1–8, Mar. 2011, doi: 10.1186/1743-422X-8-133.

[17] K. Addo *et al.*, “Knowledge, Attitudes and Practices (KAP) of Herdsmen in Ghana with respect to Milk-Borne Zoonotic Diseases and the Safe Handling of Milk,” *J. Basic Appl. Sci.,* vol. 01, no. 10, pp. 1556–1562, 2011.

[18] G. Pandey *et al.*, “Status of tuberculosis in bovine animals raised by tuberculosis infected patients in Western Chitwan, Nepal,” *Int. J. Infect. Microbiol.,* vol. 1, no. 2, pp. 49–53, Jan. 2012, doi: 10.3126/ijim.v1i2.7407.

[19] T. N. Gaire and S. Subedi, “Bovine tuberculosis In DOTS implemented area of Nepal scenario of zoonotic disease of Nepal,” 2012, Accessed: Oct. 07, 2021. [Online]. Available: https://www.amazon.com/Bovine-Tuberculosis-DOTS-Implemented-Nepal/dp/365913192X.

[20] F. Olea-Popelka *et al.*, “Zoonotic tuberculosis in human beings caused by Mycobacterium bovis—a call for action,” *Lancet Infect. Dis.,* vol. 17, no. 1, pp. e21–e25, Jan. 2017, doi: 10.1016/S1473-3099(16)30139-6.

[21] K. P. Acharya, N. Niroula, and K. Kaphle, “Retraction: Review of brucellosis in Nepal (Epidemiology and health (2016)),” *Epidemiology and health*, vol. 39. NLM (Medline), p. e2017018, 2017, doi: 10.4178/epih.e2017018.

[22] E. Lindahl, N. Sattorov, S. Boqvist, and U. Magnusson, “A study of knowledge, attitudes and practices relating to brucellosis among small-scale dairy farmers in an urban and peri-urban area of Tajikistan,” *PLoS One,* vol. 10, no. 2, pp. 1–10, 2015, doi: 10.1371/journal.pone.0117318.

[23] M. Delelegn and Y. Girma, “Assessment of community knowledge, attitude and practice on milk borne zoonoses disease in Debre- Birhan town, north Shewa, Ethiopia,” *J. Public Heal. Epidemiol.,* vol. 10, no. 4, pp. 123–131, Apr. 2017, doi: 10.5897/JPHE2017.0983.

[24] H. Çakmur, L. Akoğlu, E. Kahraman, and M. Atasever, “Evaluation of farmers’ knowledge-attitude-practice about zoonotic diseases in Kars, Turkey,” *Kafkas J. Med. Sci.*, vol. 5, no. 3, pp. 87–93, 2015, doi: 10.5505/KJMS.2015.83436.

[25] V. Rajshekhar, D. D. Joshi, N. Q. Doanh, N. Van De, and Z. Xiaonong, “Taenia solium taeniosis/cysticercosis in Asia: Epidemiology, impact and issues,” *Acta Trop.*, vol. 87, no. 1, pp. 53–60, 2003, doi: 10.1016/S0001-706X(03)00055-X.

[26] T. R. Kelly *et al.*, “Awareness and practices relating to zoonotic diseases among smallholder farmers in Nepal,” *Ecohealth*, vol. 15, no. 3, pp. 656–669, Jan. 2018, doi: 10.1007/S10393-018-1343-4.

[27] T. Chowdhury, Marufatuzzahan, P. Shanzana, and F. N. Zahan, “Knowledge, awareness, and risk of zoonotic diseases among the smallholder livestock farmers in suburban areas of Sylhet, Bangladesh,” *Adv. Biol. Earth Sci.*, vol. 3, no. 1, pp. 69–84, 2018.

[28] S.-D. Ziblim, S. B. Suara, and A. Jemilatu, “Knowledge, attitude, and practices towards zoonotic diseases among cattle farmers in rural communities in Tamale, Northern Region of Ghana,” *Int. J. Med. Res. Heal. Sci.*, vol. 10, no. 2, pp. 98–106, 2021.

[29] I. Ausraful *et al.*, “Pig raising practices by unprivileged, ethnic people in Bangladesh,” *Insights Vet. Sci.*, vol. 5, no. 1, pp. 001–005, 2021, doi: 10.29328/journal.ivs.1001028.
