# Supplementary material for: Regional variation in knowledge and practice regarding common zoonoses among livestock farmers of selective districts in Nepal
Source: PLoS Negl Trop Dis. 2023 Feb 14;17(2):e0011082. doi: 10.1371/journal.pntd.0011082 (PMC9928098; doi:10.1371/journal.pntd.0011082)
Supplement: Attachment — Submitted filename: Commenrs for PNTD-D-22-00597.docx [file pntd.0011082.s003.docx]

Reviewer comments on the Manuscript PNTD-D-22-00597

**Tittle:**

1. The title should be rewritten and the word “in” should be replaced with “among”

**Abstract:**

2. It appears like the main outcome is zoonotic disease risk and not the knowledge and practices difference as been very largely articulated in the text. If correct, the Authors needs to revise the Manuscript to bring this out clearly.
3. The methods should be rewritten to bring out study design, participants, data capture, analysis technique and the results presentations.
4. The results on the practices needs to be reported and rewritten clearly based on the study findings
5. The conclusion in the abstract is misleading, since a majority of results presented are about/over 80% and that cannot be generalized as poor understanding. This should be rewritten.
6. Also, the recommendation should be inclined onto the study objectives. This study was on prevalence. Therefore, a string recommendation should be mad on the Risk/Knowledge difference and the associated practices.

**Introduction:**

7. The first sentence, in the first paragraph needs to cited and referenced appropriately
8. The second paragraph needs to be rewritten since it does not bring out the challenge of zoonotic disease in animal husbandry properly and instead is diversionary to the general agro-production challenges
9. In the third paragraph; the authors should rewrite and paraphrase the grammar.

**Methods and Material:**

10. Appropriate map of the study are should be provided to support the study locations and ecological zones arguments
11. Provide the sample size calculation formular that was used
12. Why are the perceptions results not included in the present study??
13. Please provide a clear data analysis technique(s), including the Softwares that were used

**Results:**
17. All results must have appropriate headings (tittle) e.g; Social demographic characteristics of the study participants

14. All tables should be named appropriately; e.g: “Table 1 showing…….”
15. Results on the knowledge sections should be separated in the sub-sections and represented appropriately
16. The authors need to clearly state the test statistics being represented in the different tables, and should avoid biasing the readers/reviewers by simply showing the p-value in the different tables

**Discussion:**

17. The discussion is too lengthy. Needs to be downsized, cited and referenced appropriately

**General:**

18. Throughout the document, the authors kept on mentioning the six zoonotic diseases. This has no background in the general write-up; the authors should consider strengthening these
19. The introduction section needs to rewritten to bring out the inverted pyramid approach
20. Other supplementary materials if available should be presented and published along with the findings to back up the study results

**Decision:**

21. Should be published when comments are made
