# Supplementary material for: Regional variation in knowledge and practice regarding common zoonoses among livestock farmers of selective districts in Nepal
Source: PLoS Negl Trop Dis. 2023 Feb 14;17(2):e0011082. doi: 10.1371/journal.pntd.0011082 (PMC9928098; doi:10.1371/journal.pntd.0011082)
Supplement: Attachment — Submitted filename: Response to Editor and Reviewers.docx [file pntd.0011082.s004.docx]

**Response to Editor and reviewers**

Dear editor and reviewers,

Thank you very much for worthy comments we have now revised the manuscript as per your comments and suggestion. We hope the manuscript is much improved than previous one. Your suggestions and comments will be highly appreciated, please let us to know if further revision required. Once again thank you very much for your support. Please find the comments and response matrix below.

Thank you!

Reviewer comments on the Manuscript PNTD-D-22-00597

| S.N. | | Comments | | Response | | Remarks |
| --- | --- | --- | --- | --- | --- | --- |
| **Reviewer- 1** | | | | | | |
| **Title:** | | | | | | |
| 1. | | The title should be rewritten and the word “in” should be replaced with “among” | | Thank you. We have replaced the word ‘in’ by ‘among’ in the title. | |  |
| **Abstract:** | | | | | | |
| 2. | | It appears like the main outcome is zoonotic disease risk and not the knowledge and practices difference as been very largely articulated in the text. If correct, the Authors needs to revise the Manuscript to bring this out clearly. | | Thank you. We have revised the manuscript. | |  |
| 3. | | The methods should be rewritten to bring out study design, participants, data capture, analysis technique and the results presentations. | | Thank you. We have re-written methodological section in both abstract and main part. | |  |
| 4. | | The results on the practices needs to be reported and rewritten clearly based on the study findings. | | Thank you. We have re-written based on findings. | |  |
| 5. | | The conclusion in the abstract is misleading, since a majority of results presented are about/over 80% and that cannot be generalized as poor understanding. This should be rewritten. | | Thank you. We have revised conclusion in abstract section based on study findings. | |  |
| 6 | | Also, the recommendation should be inclined onto the study objectives. This study was on prevalence. Therefore, a string recommendation should be mad on the Risk/Knowledge difference and the associated practices. | | Thank you. We have recommended some interventional programs based on research findings. | |  |
| **Introduction:** | | | | | | |
| 7 | | The first sentence, in the first paragraph needs to cited and referenced appropriately. | | Thank you. We have added citation in first sentence in the first paragraph. | |  |
| 8 | | The second paragraph needs to be rewritten since it does not bring out the challenge of zoonotic disease in animal husbandry properly and instead is diversionary to the general agro-production challenges | | Thank you. We have re-written second paragraph. | |  |
| 9. | | In the third paragraph; the authors should rewrite and paraphrase the grammar. | | Thank you. We have re-written third paragraph supported by English editor. | |  |
| **Methods and Material:** | | | | | | |
| 10. | | Appropriate map of the study are should be provided to support the study locations and ecological zones arguments. | | Thank you. We have added appropriate map, which indicated study areas. | |  |
| 11. | | Provide the sample size calculation formular that was used. | | Thank you. We now mentioned the sample calculation formula. | |  |
| 12. | | Why are the perceptions results not included in the present study?? | | Thank you. Based on our research objectives we only pick knowledge and practice related data and exclude the perception related data in the study. | |  |
| 13. | | Please provide a clear data analysis technique(s), including the Software that were used | | Thank you. We have mentioned clearly about the data analysis techniques including appropriate software. | |  |
| **Results** | | | | | | |
| 14. | | All tables should be named appropriately; e.g: “Table 1 showing…….” | | Thank you. We have re-written all table heading. | |  |
| 15. | | Results on the knowledge sections should be separated in the sub-sections and represented appropriately | | Thank you. We have mention separate heading related to zoonoses related knowledge, i.e., knowledge on zoonotic rabies etc. | |  |
| 16. | | The authors need to clearly state the test statistics being represented in the different tables, and should avoid biasing the readers/reviewers by simply showing the p-value in the different tables | | Thank you.  We have mentioned the test statistics used for measuring association in the table. | |  |
| 17. | | All results must have appropriate headings (tittle) e.g; Social demographic characteristics of the study participants. | | Thank you. We have mention results section under the appropriate heading. | |  |
| **Discussion:** | | | | | | |
| 18. | | The discussion is too lengthy. Needs to be downsized, cited and referenced appropriately | | Thank you. We have tried to downsize with appropriate citation. | |  |
| **General:** | | | | | | |
| 19. | | Throughout the document, the authors kept on mentioning the six zoonotic diseases. This has no background in the general write-up; the authors should consider strengthening these | | Thank you. Among the 11 common zoonotic disease, which declared by Asian Network for Sustainable Agriculture and Bioresources (ANSAB), researchers limited only six common zoonoses in this study, which are closed to livestock. We have mentioned those things under the heading of inclusion criteria. | |  |
| 20. | | The introduction section needs to rewritten to bring out the inverted pyramid approach | | Thank you. We have re-written. | |  |
| 21 | | Other supplementary materials if available should be presented and published along with the findings to back up the study results | | Thank you. | |  |
| **Decision:** | | | | | | |
| 22. | | Should be published when comments are made | | Thank you. We have now addressed all comments. We have tried our best to response the comments. | |  |
| **Reviewer - 2** | | | | | | |
| **S.N.** | **Comments** | | **Response** | | **Remarks** | |
| 1. | The authors have declared that no competing interests exist. | | Thank you. We had re-written with details information. | |  | |
| 2. | If it is possible some extra details should be added- ex. study design. | | Thank you. We had mention more details in this section. | |  | |
| 3. | How these diseases are chosen? | | Thank you. Among the 11 common zoonotic diseases in Nepal, which declared by Asian Network for Sustainable Agriculture and Bioresources (ANSAB), we have included only six common zoonoses in this study, which are closed to livestock. We have mentioned those things under the heading of inclusion criteria. | |  | |
| 4. | I would specify that these are regions | | Thank you. Geographically, Nepal divided in three parts, Mountain, Hill and Terai. The altitude of the terai region is from 60 metres to 610 metres whereas the altitude of hilly region is from 610 metres to 4877 metres from the sea level. Similarly, the altitude of the mountain region is from 4877 metres to 8848 metres. In this article authors tried to determine regional variation on zoonoses related knowledge and preventive practices that help for determine the zoonoses risk in farming communities in Nepal. | |  | |
| 5. | Reference | | Thank you. We have added. | |  | |
| 6. | Reference | | Thank you. We have added. | |  | |
| 7. | Map | | Thank you. We have added appropriately. | |  | |
| 8. | More details | | Thank you. We have followed the Delphi technique during the tools design. It is effective technique to maintain the reliability of research tools. | |  | |
| 9. | Details | | Thank you. We had mention detailly. | |  | |
| 10. | Copy of the survey? | | Thank you. This article is part of PhD dissertation of first authors. So, almost data was used from the dissertation. Verbal consent was taken from the participants due to the COVID-19 pandemic; data was collected maintaining standard protocol made by WHO and Ministry of Health and Population, Nepal. | |  | |
| 11. | Only disease without definition. | | Thank you. We have added. | |  | |
| 12. | Acronym not introduced before | | Thank you. We have revised. | |  | |
| 13. | Reference | | Thank you. We have added. | |  | |
| 14. | Source? Maybe introduce these details before, together with the explanation of why it is included | | Thank you. We had revised the sentence with citation. | |  | |
| 15. | Source | | Thank you. We added it. | |  | |
| 16. | Not introduced TB and BTB | | Thank you. We have re-written. | |  | |
| 17. | From the table, it is difficult to understand if the question is:  1) pregnant women exposure to livestock  2) pregnant women with a role of taking care  they can be exposed to livestock without taking care of them ex proximity. | | Thank you. We have re-written. | |  | |
| 18. | If possible, add more references "several studies" | | Thank you. We have re-written. | |  | |
| 19. | The parenthesis does not close | | Thank you. We have re-written. | |  | |
| 20. | For the bovine TB the result was put into a nice context, if it is possible, I would do the same for each disease to show its relevance and how is Nepal compare to the rest. | | Thank you. | |  | |
| 21 | I would put it before, in the discussion do not support your point | | Thank you. We have removed it. | |  | |
| 22. | Same comment than above - more context will strengthen the comparison | | Thank you. | |  | |
| 23 | Reference | | Thank you. Added. | |  | |
| 24 | No need to restate it [PEP] | | Thank you. We removed PEP. | |  | |
| 25 | General overlaps with the result section. the reader could get overwhelmed - what are the main key messages to bring home? | | Thank you. We have re-written. | |  | |
| 26 | limitation and future plans need to be explicit | | Thank you. We have mentioned limitation and further plan in separate heading before the conclusion section. | |  | |
