# Supplementary material for: Regional variation in knowledge and practice regarding common zoonoses among livestock farmers of selective districts in Nepal
Source: PLoS Negl Trop Dis. 2023 Feb 14;17(2):e0011082. doi: 10.1371/journal.pntd.0011082 (PMC9928098; doi:10.1371/journal.pntd.0011082)
Supplement: Attachment — Submitted filename: Response to Reviewers.docx [file pntd.0011082.s005.docx]

**Response to Editor and Reviewers**

Dear Editor and Reviewers,

Thank you very much for your worthy comments we have now revised the manuscript as per your comments and suggestion. We hope the manuscript is much improved than the previous one. Your suggestions and comments will be highly appreciated, please let us know if further revision is required. Once again thank you very much for your support. Please find the comments and response matrix below.

Thank you!

Reviewer comments on the Manuscript PNTD-D-22-00597

| S.N. | | Comments | Response | Remarks |
| --- | --- | --- | --- | --- |
| **Reviewer- 1** | | | | |
| **Comments in** [Results and discussion section] | | | | |
| 1. | | Some repetitions between the results and the discussion part slow the flow of the manuscripts and result in a lengthy discussion. | Thank you. Now we have revised the manuscripts as per the comments. |  |
| 2. | |  | We removed some sentences from the results section [from knowledge about bovine tuberculosis] | Last few sentences on Pages 16 and 17 |
| 3. | | Some parts of the discussion would benefit from references to support the claim. | Thank you. We have added some citations/references to the discussion. | References no 25 and 38 |
| 4. | | Some parts of the discussion would also inconsistencies in how acronyms are introduced in the manuscript used. Therefore, it is advised to spell out the words before using the acronym and to be consistent with the same acronym (for example, Bovine TB vs BTB) | Thank you. We have revised the discussion section with an appropriate acronym.  We have used Bovine tuberculosis except for BTB or Bovine TB. |  |
| # | **General** | |  |  |
|  | Authors summary: | | We have revised the author summary as per the journal format. |  |
|  |  | | We changed the word geographical region to the word ecological region throughout entire the article. We felt the word ecology is more sensible than geographical. |  |
|  |  | | English language and other minor corrections were performed necessarily. |  |
